# Detection Methods of Human and Animal Influenza Virus—Current Trends

**DOI:** 10.3390/bios8040094

**Published:** 2018-10-18

**Authors:** Karolina Dziąbowska, Elżbieta Czaczyk, Dawid Nidzworski

**Affiliations:** 1 Institute of Biotechnology and Molecular Medicine, 3 Trzy Lipy St., 80-172 Gdansk, Poland; ela@etongroup.eu (E.C.); dawid@etongroup.eu (D.N.); 2 SensDx SA, 14b Postepu St., 02-676 Warsaw, Poland

**Keywords:** influenza virus, Rapid Influenza Diagnostic Tests RIDTs, electrochemical detection, optical detection, ELISA, PCR

## Abstract

The basic affairs connected to the influenza virus were reviewed in the article, highlighting the newest trends in its diagnostic methods. Awareness of the threat of influenza arises from its ability to spread and cause a pandemic. The undiagnosed and untreated viral infection can have a fatal effect on humans. Thus, the early detection seems pivotal for an accurate treatment, when vaccines and other contemporary prevention methods are not faultless. Public health is being attacked with influenza containing new genes from a genetic assortment between animals and humankind. Unfortunately, the population does not have immunity for mutant genes and is attacked in every viral outbreak season. For these reasons, fast and accurate devices are in high demand. As currently used methods like Rapid Influenza Diagnostic Tests lack specificity, time and cost-savings, new methods are being developed. In the article, various novel detection methods, such as electrical and optical were compared. Different viral elements used as detection targets and analysis parameters, such as sensitivity and specificity, were presented and discussed.

## 1. Introduction

From humanity’s common illnesses, the most frequent are acute respiratory infections (ARIs). Influenza plays a role of the most serious virus causing ARIs and is the most often detected in lung infections. Other causatives of ARIs can be rhinovirus, parainfluenza, adenovirus, enterovirus, respiratory syncytial virus (RSV) and others [1,2].

Influenza wears the name of ‘the mother of all pandemics’. In particular, influenza A type has the ability to cause worldwide epidemics. In the last hundred years, virus attacks were documented four times: The Spanish flu (H1N1) in 1918, Asian flu (H2N2) in 1957, Hong Kong influenza (H3N2) in 1968 and swine influenza (H1N1) in 2009. It seems obvious to expect an influenza pandemic to return [3,4]. The illness affects groups of all ages, and this pattern is not common for most viruses. There is a high possibility for every human to suffer from influenza. Groups infected are mostly schoolchildren, the elderly and patients with serious medical conditions. Infections of respiratory viruses, mainly influenza, and RSV, occur seasonally in winter months (from December to March) in Europe, what is less common in hot climates where rhinovirus is seen in fall and spring, while adenovirus infects all year [2]. Influenza spreads very easily among the population and shows high attack rates. In the US, it is the 8th highest cause of death, infects around 0.4 to 0.6 billion of children and 0.2 to 5.0 billion adults worldwide [5], kills 0.5 to 1 million people annually with numbers still increasing [6,7].

The influenza virus construction is comparatively simple. It mainly contains 8-segmented RNA and surface proteins with highly immunogenic properties. Distinguishable are three types—influenza A, B, and C, all belonging to the *Orthomyxovirus* family. The A-type is mostly responsible for pandemics in the 20th–21st century. Two glycoproteins cover the viral surface: haemagglutinin (HA) and neuraminidase (NA) in a ratio of four to one [8]. Based on surface composition, distinguishable are 18 H (H1–H18) and 11 N (N1–N11) subtypes forming potentially 198 combinations [3]. Influenza A infects the human population, birds, pigs, dogs, horses and more [9]. Genetic recombination is possible through the segmented genome. Reassortment of genes is highly important in the epidemics. Human population does not have the immunity against mutants with new HA and NA antigens on the virion surface. There is a possibility of interspecies transmission without genetic reassortment, like in the case of H1N1 virus between swine and humans (and conversely) or H9N2 from poultry to humans. In other cases, RNA segment reassortment occurs if at least two influenza viruses infect a single hosts cell [10,11]. Animal influenza viruses which occasionally infecting humans are called zoonotic influenza viruses (in direct and indirect contact) [12]. This high possibility of genetic variation can have subsequently pandemic effects. Most of the new influenza viruses are mutants forming from antigenic drift [11].

The B-type influenza virus has similar biological properties to the A-type one. However, through electron microscopy, they are indistinguishable in size and shape. Influenza B infects mainly humans and rarely other species. The antigenic drift occurs less often than in the A-type virus [8,9].

The C-type influenza virus naturally infects humans but is less frequently detected, causes mild pediatric infections and sometimes affects adults [13]. It differs from A and B types through a shorter genome (1 segment less), and its major surface glycoprotein is hemagglutinin-esterase-fusion (HEF), functioning as H and N together [14]. Additional minor protein M2 is categorized as a single-pass integral membrane protein. It plays the role of a proton-selective ion channel, pH sensitive [15].

Recently, new influenza virus genus was isolated from pigs and cattle and specified as D virus. It shows many similarities to C type virus. However, its structural differences make it a danger to public health due to the ability of binding human tracheal epithelia [9]. Some studies have shown that 94–97% of workers exposed to cattle breeding have specific antibodies against influenza D, what means a risk of zoonotic infections. Real-time polymerase chain reaction (RT-PCR) assay is believed to be adequate for influenza D virus infection diagnosis [16].

## 2. Influenza Pathogenesis

The influenza virus has a diameter of around 100 nm [17]. Influenza A virus proteins (HA, NA, and M2) are localized externally on the surface, more specifically they protrude above the lipid membrane. The infection starts with virus linking to the host respiratory epithelial cells. It recognizes and binds to sialic acid receptors via H proteins.

Sialic acids are nine-carbon acidic monosaccharides mainly found at the end of many glycoconjugates. The terminal carbon-2 can bind to carbon-3 or carbon-6 of galactose, forming different α-linkages and steric configurations. In human population dominate α-2,6 bonds, while α-2,3 are also common; however, the latter are more common in other species (ducks, birds). So there is a possibility of human infection by avian influenza, but less efficiently [18,19]. The next step is neuraminidase activity. Sialic acid is rifted from the cell’s surface, what enables the influenza virus release and distribution in the respiratory tract. The NA protein plays a role in replication of A and B influenza types. The M2 protein is essential after cell entry through uncoating of influenza A virus [13]. Influenza incubation lasts 1 to 4 days, then the virus sheds and symptoms appear. Viruses circulate in a host for 5 to 10 days but decrease 3–5 days after the first symptoms [20].

Viral RNA genome is segmented thus recombination between different strains is possible. This process is called antigenic shift; however, it is sporadic and occurs less than once per decade [3]. As a result, surface glycoproteins undergo some variations (minor changes in amino acid sequence like point mutations in genes). The ability of influenza virus to progressive antigenic change forces updates of vaccines composition [21]. Influenza glycoproteins are an excellent target for virus detection due to many copies of HA (around 300) and NA (around 50) on one viral particle [21,22]. Also, the nucleoprotein (NP) of influenza differs between A and B types and is often a target in antigen-detection type tests [21].

## 3. Influenza Diagnosis

Influenza is a contagious viral respiratory disease caused by the influenza virus. However, many respiratory viruses and bacteria can give similar symptoms and cover up a serious infection. Examples are Chlamydia, Legionella, and Mycoplasma. Thus, it is difficult to distinguish different pathogens from influenza basing only on clinical features [13].

Inactivated influenza virus vaccines (IVV) are the primary method for influenza prevention and are being used for over 60 years [23]. Vaccines are updated annually to match current strain trends, as antigenic drift frequently occurs in A and B influenza viruses [24]. However, they provide only moderate protection, especially for children and the elderly. This can be caused by preclinical tests on animals which differ in immunocompetence or immune history compared to humans. Deviations are also dependent on vaccines formulation, which can contain DNA, peptides, recombinant proteins and others. Rowell et al. [25] confirmed the influence of immune history on vaccine protection level. The traditional influenza vaccine was inserted into mice after two pre-vaccinations with different vaccine types. Immune responses were examined by enzyme-linked immunosorbent assay (ELISA) and interferon-γ enzyme-linked immunospot assay (ELISPOT). Results showed that traditional vaccine protection was enhanced, unchanged or negatively interfered when prior vaccination was used.

Despite the fact of vigorous scientists’ work on influenza prevention, there is still a huge number of human deaths, thus fast diagnostic methods may be the salvation. The greatest treatment benefits can be obtained when medicine is given within the first 24–30 h of first infection symptoms [26]. Moreover, antivirals lose their efficacy in 24 to 48 h in cases of people with a weak immune system [27]. Nowadays, significant interest is focused on developing devices able to detect various diseases in an easy and reliable way, within short time and at the early stage of the infection. The main goal is to enhance the efficiency of a treatment [26]. However, it is challenging to design molecular-based diagnosis tests when influenza characterizes with extreme genetic variability. It is important to understand pathogen action in the host organism exactly and precisely follow infection and post-infection reaction mechanisms.

## 4. Influenza Virus Conventional Detection Methods

Influenza detection methods can be divided into two types: traditional and novel. Classic methods are known for many years and are possible to perform under standard laboratory conditions or by stripe-type tests and are presented in Table 1.

The basis of the traditional studies is genetic analysis with two main ‘gold standard’ assays: reverse transcription- polymerase chain reaction (RT-PCR) and ELISA. Currently, the fast diagnostic tests for influenza dominate the market. Generally, they include (except mentioned above) serology, viral culture, rapid antigen testing, immunofluorescence assays, and others. The specificity and sensitivity vary by the test type, performing laboratory, specimen type and more. It is believed that nasopharyngeal swabs have a higher yield than nasal or throat ones in rapid influenza detection [28]. Many specialists still ensure that during influenza season respiratory samples should be tested by all methods: viral culture, molecular assay, and RIDTs for disease detection and for controlling of new mutant strains that may include in next vaccine sort.

### 4.1. Rapid Influenza Diagnostic Tests (RIDTs)

**Figure 1:**
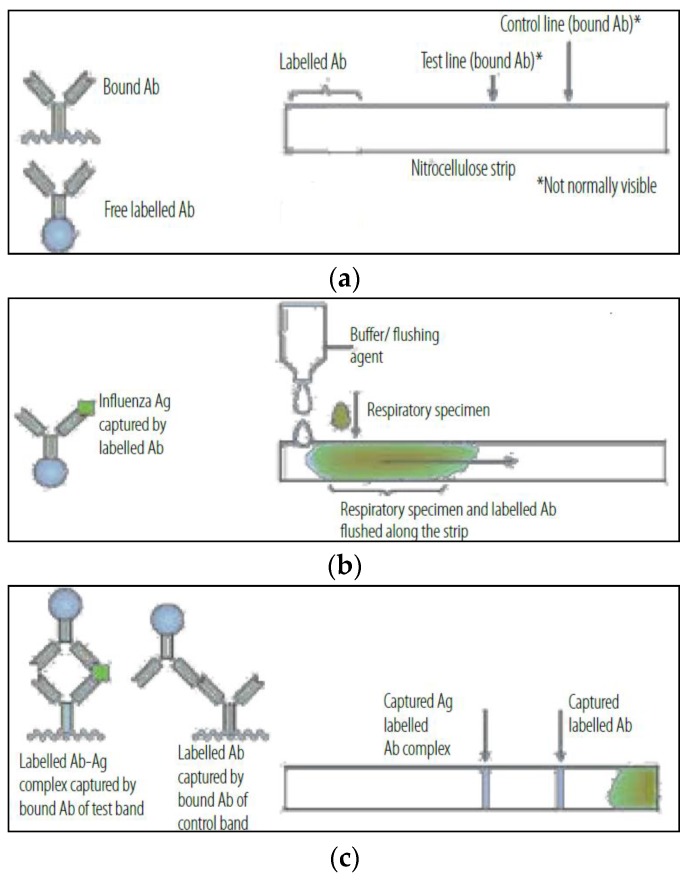
RIDT mode of action: (**a**) Bounding dye-labeled antibodies specific for target antigen onto the nitrocellulose strip; (**b**) Addition of respiratory specimen with buffer to the strip; (**c**) Trapping antibodies on the test line if target antigen is presented. Reproduced with permission from WHO on behalf of the Special Programme for Research and Training in Tropical Diseases, Use of Influenza Rapid Diagnostic Tests; published by WHO Library Cataloguing-in-Publication Data, 2010.

The ‘gold star’ are commercial RIDTs showing results in around 15 min. RIDTs are immunoassays that can identify influenza viral nucleoprotein antigens in respiratory specimens. Monoclonal antibodies target viral nucleoprotein using immunochromatographic or immunoassay techniques. Observed results are color changes or some other optical signals [29]. For example ‘Alere and Influenza A&B’ test uses an isothermal nucleic acid amplification, and ‘BD Veritor’ uses chromatographic techniques as influenza detection methods. The mode of action of RIDT is presented in Figure 1.

The main advantage is the possibility of providing the test in a physician’s office in an effortless way with approximately moderate sensitivity (50–80%) [30] and high specificity (90%), but only in a qualitative way (positive/negative). Thus, false positive and false negative results should be considered, especially during high influenza activity season [29]. False-negatives occur more often than false-positives. Negative results cannot exclude virus infection [31]. Undeniably, this kind of tests contributes to shortening the time of infection diagnosis, especially when the algorithm of influenza detection is long and laboratory procedures and requirements are high [32,33]. RIDTs performance is better in children than adults (around 13% greater) due to higher viral loads and longer viral shedding [34]. Tests differ in the virus type detected and with distinguishing between types, it means detection of only A type, A and B types with or without distinguishing between them [35]. Sensitivity for B-type virus detection is lower than A-type. None of RIDTs can distinguish influenza A subtypes. In some tests, the results standardization is possible due to analyzing devices. They are available in cassette, card and dipstick formats, with a visual inspection or automated readers [36]. RIDTs belong to II class of devices which need general and special controls. They were reclassified by Food and Drug Administration (FDA) from I class with low risk due to many failures in the H1N1 pandemic in 2009. Also, FDA evaluated some criteria for RIDTs that must be adhered [31].

As the market is enriched in commercial RIDTs, many authors have compared their sensitivities and specificities in influenza A/B detection. Ryu et al. [34] have compared three digital RIDTs ‘Sofia Influenza A + B Fluorescence Immunoassay’, ‘BD Veritor System Flu A + B assay’ and ‘BUDDI Influenza A and B test’ with conventional ‘SD Bioline’. Results have shown for influenza A: BUDDI, Sofia, Veritor, and Bioline sensitivity on level 87.7%, 94.5%, 87.7%, and 72.6%, respectively and specificity: 100%, 97.7%, 96.5%, and 100%, respectively, which are only partially accurate with producers assurance. For influenza B, sensitivities and specificities were on similar levels. None of the tests showed cross-reactivity with other respiratory viruses. The Sofia test with the fluorescence reader had the best sensitivities. RIDTs with digital readout systems showed many similarities to conventional assays like small sample volume (less than 150 µL) and short analysis time (around 15 min) but exhibited much better sensitivities, even one order of magnitude lower limits of detection (LODs).

‘GOLD SIGN FLU’ and ‘Quick Navi-Flu’ two RIDTs basing on immunochromatography were compared by Akaishi et al. [37] They were applied for nasopharyngeal swabs as appropriate for the influenza A and B antigens detection. Quick Navi-Flu demonstrated better sensitivities and specificities. Advanced were short detection time (less than 10 min for most probes) compared to fluorescence-based tests.

Another type of point-of-care (POC) test ‘the cobas^®^ Liat^®^ Influenza A/B’ was evaluated by Melchers et al. [38] The molecular assay based on RT-PCR showed results in 20 min. Detection time of immunofluorescent pads was shorter, but sensitivities varied from 50% to 70% and often had to be confirmed by PCR methods. The cobas^®^ Liat^®^ longer analysis time was balanced with an automated system, higher sensitivities (96%) and specificities (100%) compared to another test (Diagenode). This assay was faster than others FDA-approved RT-PCR-based tests with analysis time ≥1 h.

Two authors evaluated and compared different RIDTs on a wide range of patients for influenza and RSV detection and distinction. Gómez et al. [39] tested 209 breath samples (nasopharyngeal swabs or aspirates) from both adults and children and Moesker et al. [40] used >500 swabs only from pediatric patients (aged 0–5 years). Fluorescence immunoassays (FIAs) with europium dye ‘Sofia^®^ Influenza A + B’ and ‘Sofia^®^ RSV’ were compared to reference methods, RT-PCR, and cell culture. Automatic analyzers showed higher sensitivities and specificities than other market RIDTs [39]. Comparison of ‘Influenza AB^®^’ and ‘BinaxNow RSV^®^’ tests with a traditional RT-PCR confirmed the lower accuracy of RIDTs than in virus isolation and RT-PCR methods [40]. Pediatric specimens were showing higher sensitivities as children have higher viral titers thus better RIDTs responses.

In the literature, RIDTs evaluation or comparison to each other or the reference points are trending in the last few years. Besides the examples described above, available are also ‘Alere i Influenza A&B’ nucleic acid amplification versus ‘Xpert Flu/RSV’ [41], ‘the BD Veritor™ System Flu A + B’ versus the ‘SD Bioline assay’ [34], mariPOC^®^ test [42], Becton Dickinson test [43], ‘FLU A + B’ vs real-time-PCR [44], and many more [45,46,47,48,49,50,51], focusing on saliva specimens or nasopharyngeal swabs [52].

### 4.2. Immunofluorescence Assays

Immunofluorescence method is an antigen detection with fluorescent microscope usage. The results are received in 2–4 h with high specificity and moderate sensitivity. Distinguishable are direct (DFA) and indirect (IFA) fluorescent antibody staining assays for influenza A and B detection. Subtyping of influenza A virus is not possible [53]. DFA assays are believed to have an easy procedure and short response time, so they are popularly used for influenza diagnosis. In the test, respiratory epithelial cells from nasopharyngeal swabs are directly stained with fluorescent-labeled antibodies and examined under fluorescent microscope. Sensitivities are around 60–80% levels. On the USA market, only two approved tests by FDA can be found, which are the D3 FastPoint L-DFA and Bartels Viral Respiratory Screening and Identification Kit [54]. DFA tests do not win in the influenza diagnosis with molecular assays which are giving higher sensitivities.

### 4.3. Serological Assays

From serological tests, the most commonly used are hemagglutination inhibition assay (HAI), microneutralization/virus neutralization assay (VN), single radial hemolysis (SRH), complement fixation assay, enzyme-linked immunosorbent assay (ELISA) and Western blotting [55]. These kinds of tests are generally not recommended because of paired serum samples necessity. The first swab must be collected as soon as possible at the beginning of an infection and the second about 2–4 weeks after. There is also a problem with the test availability. The results from a single serum specimen are not interpretable. Although the assay is cheap and simple, the sensitivity is unsatisfactory [28,53].

HAI is generally used for influenza antibodies detection, which inhibits the interaction between H glycoprotein and red blood cells receptors. The test can be performed on inactivated viruses and is positive when four-fold or more rise of specific antibody titer is observed. The rise is between acute and convalescent serum samples and is measured by hemagglutination inhibition [55].

In the SRH method, the formed complex of antigen-antibody induces the measurement of hemolysis areas which are proportional to antibodies quantity. There is no pretreatment of the serum needed. The technique is commonly used in natural infections and vaccinations [56]. The SRH gives higher sensitivities than the HAI assay.

The VN measures virus-specific antibodies induction and their ability for virus neutralization. As a result, viral cells infection is prevented. This method is also routinely used in natural infections and vaccinations in influenza seasons. The VN assay gives higher sensitivities than the HAI assay, but there are some restrictions in the diagnostic application (certified laboratories necessity). This assay requires infectious, active viruses [55].

ELISA tests [57] are performed since the 1990s with high sensitivity and specificity. They are available in two forms: paper strips and microtiter plates. Despite the big popularity of the test, the major disadvantage is still the lower sensitivity compared to tests based on nucleic acid- techniques. In a conventional test, the influenza virus is detected through specific antigen-antibody interaction and immunocomplex- enzyme linkage, resulting in color change [58]. Some research groups are working on enhancing the sensitivity of these tests by using gold and europium nanoparticles with positive results. The europium nanoparticle-based immunoassay (ENIA) detects 29 strains of influenza A virus and some B virus subtypes with 16-times higher sensitivity than commercial ELISA assay [59].

### 4.4. Cell Culture Based Detection

The Viral Culture method was introduced in the 1940s and is believed to be the most traditional and the gold standard for influenza diagnosis [31]. Influenza viruses are recovered in clinical samples through propagation in mammalian cells or embryonated eggs. The principle is inoculation of permissive cell lines or embryonated eggs with infectious samples, propagation for a week (up to 10 days), observation of the cytopathic effect, and checking virus infection by various methods: immunofluorescence microscopy, antibody staining or erythrocytes hemadsorption [54].

### 4.5. Nucleic Acid-Based Tests (NATs)

These methods base on PCR and detection of specific DNA/RNA sequences of the virus. NATs offer higher sensitivity than antigen-based tests and in much shorter time. Currently available are reverse transcriptase-PCR (RT-PCR), sequencing-based tests like Next-Generation Sequencing (NGS), ligase chain reaction, DNA microarray tests, simple amplification-based assay (SAMBA), nucleic acid sequencing-based amplification (NASBA), loop-mediated isothermal amplification-based assay (LAMP) and more. General majority of NATs is performed within 2–4 h with influenza A subtypes information. Commercially available are 26 FDA licensed NATs for influenza virus detection [60].

RT-PCR allows identification of influenza viral RNA in respiratory specimens and is believed to be the most powerful influenza identification assay all over the world. It uses nested primers to detect and subtype influenza viruses [13]. Results of the analysis offer very high specificity and the sensitivity is believed to be the highest of all conventional detecting methods [31]. The test procedure contains viral RNA extraction from a specimen, RNA reverse transcription to single-stranded complementary DNA (sscDNA) by reverse transcriptase enzyme and product amplification with fluorescent detection. Some molecular assays using RT-PCR technique cannot only distinguish A and B types of influenza viruses but even identify specific seasonal influenza A subtypes, like H1N1 or H3N2 [61]. RT-PCR method compared to cell culture and ELISA shows 103- and 106-times higher sensitivity, respectively. Additionally, this sensitivity is not dependent on the patients’ age. The procedure of combining multiple primers sets in ‘multiplex RT-PCR’ method enables detection of several respiratory viruses in one reaction. The main disadvantage is the 1–8 h long reaction time and diagnosis costs, as RT-PCR is the most expensive test kind [13,60]. Except conventional RT-quantitative PCR (RT-qPCR), specialists are working on speeding up the analysis, like one-step high-speed droplet-RT-PCR, getting results within 14 min [62].

SAMBA method involves isothermal nucleic acid amplification with the three-step procedure: viral RNA extraction, DNA amplification by isothermal DNA polymerase and dipstick system detection. Results are obtained in around 2 h. This test is appropriate for avian and human seasonal influenza and gives high sensitivity (100% and 97.9% for influenza A and B) [63].

NASBA is an isothermal amplification assay, PCR-independent. In one reaction it uses three enzymes: RNAse H, T7 RNA polymerase, avian myeloblastosis virus reverse transcriptase (AMV-RT). Single-stranded DNA probes are used to capture viral RNA sequences, then separated on a microfluidic chip. The test is suitable for seasonal influenza, in outbreaks of ARIs. Moore et al. [64] showed 100% sensitivity on 19 clinical samples. This method is applicable for detecting other viruses, like HIV, RSV, SARS [30].

LAMP approach is used for the detection of many viruses like SARS, adenovirus, rhinovirus, influenza virus and others. It uses DNA polymerase or RNA reverse transcriptase and two sets of primers which can recognize six distinct regions in viral complementary DNA (cDNA) [30]. The target gene is amplificated and determined by photometrical methods (color change when SYBR green added). The sensitivity is similar to RT-PCR assay [65]. RT-LAMP variety proposed by Parida et al. [66] showed a ten times higher sensitivity compared to an RT-PCR method, what is 0.1 TCID_50_/mL (tissue culture infection dose at 50% end point).

### 4.6. Next-Generation Sequencing Based Methods

NGS is one of the most influencing techniques in genetic and medicine fields. NGS compared to the original Sanger sequencing extremely speeds up the analysis due to automation possibilities. The next advantage is decreasing the costs from $100 million for human genome sequencing in 2001, by Sanger method, to $2400 nowadays. Some other technique, Illumina Platform, offers one million bases sequencing for the cost from $0.05 to $0.15 [67,68]. In general, Illumina Platform is basing on the amplification of nucleic acid fragments on solid substrate or bridge amplification. Whitehead et al. [69] applied Illumina NGS platform to create sequence-function map to optimize influenza-binding proteins. This technique has big chances to develop new effective influenza inhibitors. Another Platform, Roche 454 Life Sciences, developed NGS platforms based on pyrosequencing. It has found application in influenza A case for molecular markers identification, gene coding M2 protein mutation identification or Single Nucleotide Polymorphism detection in the gene coding hemagglutinin [54]. Except listed above, there are more NGS Platforms, like Pacific Bioscience, Ion Proton, Complete Genomics and more, which use different sequencing techniques and have both advantages and limitations, vary on costs, read length, analysis time and error rate. For example, the Chembio portable lateral flow platform can be performed outside the laboratory giving results in 25 min. The Luminex analysis requires fewer reagents, offers fewer errors and a wide linear range [55].

Among methods mentioned, general diagnostic tests for influenza base on virus culture (conventional and shellvial), detection of viral nucleic acid (PCR) or antigens (by neuraminidase enzymatic activity, fluorescent antibody or enzyme/optical immunoassay) and serologic tests. From them, culture methods can be excluded due to specialized laboratory requirement. Also, serologic tests are impractical, need two adequate specimens and are time-consuming. Rapid results can only give detection of nucleic acids or specific viral antigens and promote practical and useful diagnosis [21]. However, RT-PCR is still considered as time-consuming and expensive, and the ELISA test does not offer high sensitivity [70,71].

## 5. Novel Detection Methods of Influenza Virus

**Figure 2:**
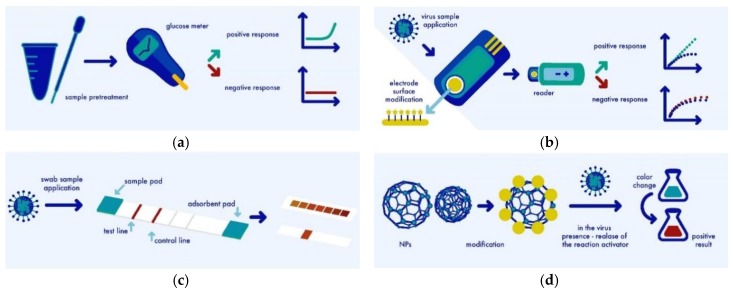
Different approaches for influenza virus detection: (**a**) Reuse of known devices like glucometers; (**b**) Electrical detection system; (**c**) Strip-based sensor/ ELISA modifications; (**d**) Optical approaches using nanoparticles.

Due to various limitations in the conventional detection methods, new diagnostic approaches are being developed. Main trends for influenza virus detection are: (I) modifications of traditional ‘gold star’ methods like PCR, RIDTs, ELISA what results in analysis time shortening, costs lowering, LOD and limit of quantification (LOQ) improvement, (II) conjugating of traditional methods and creating new platforms, micro-biochips and others, (III) introducing known solutions to new ones, like smartphone-based analysis control with results data insertion into Google Maps, (IV) reuse of the functions of known devices, like glucometer, smartphone cameras, (V) the most common used detection methods: spectral/optical, electrical, (VI) and entirely new approaches. Some of the approaches for influenza virus are presented in Figure 2. Detection limits were shown in plaque forming units (PFU), g/L, viral copies, M, hemagglutinating units (HAU), and TCID_50_ units. They were difficult to compare as relied on different quantification methods. Moreover, influenza have many components acting as target analytes, which in general are in different quantities/ratios in one viral particle or between influenza types/subtypes. Exemplary are DNA/RNA (quantified in copies/mL or M), HA (quantified in HAU) or whole viral particles (quantified in PFU). Comparison of most LODs between newly developed biosensors and different conventional methods were given.

### 5.1. Microchip Approaches

Small, micro-size devices are trending in Point of Care (POC) tests. Electromechanical systems on micro- or nano-scale have chemical, biological and medical applications. This kind of assays gives high efficiencies, small amounts of used materials and low waste production. They also speed up the analysis time and makes influenza diagnosis laboratory-independent [72]. Another critical feature is the devices’ portability. For example, using microfluidic RT-PCR with a continuous-flow and disposable electrical printed (DEP) chips an influenza virus of swine-origin can be detected in a 15 min analysis [73]. CombiMatrix Corporation developed influenza A microarray detecting all known so-far virus subtypes in less than 5 h [74]. Biochips are common. not only for influenza disease but, i.e., for tobacco mosaic virus (TMV), human rhinovirus serotype 2 (HRV2) and others [75].

### 5.2. Reuse of Known Devices

The invention of user-friendly techniques brought measurements to smartphone systems. The study of Yeo et al. [76] showed the performance of a smartphone-based rapid fluorescent diagnostic system (SRFDS) created for the H9N2 virus diagnosis in chickens. The authors used oropharyngeal (OP) and cloacal (CL) samples and compared their method with real-time RT-PCR. The limit of detection of SRFDS was 7.5 PFU/mL, what is 138-fold higher than in conventional colloidal-gold-based avian influenza rapid diagnostic test. The specificity was 100% and the sensitivity 99.44% for OP and 95.23% for CL specimens, making this test comparable to rRT-PCR. Pretreatment swab was transferred to the sample pad, after 15 min reaction time the smartphone camera was used as a detector, by using filtration of the excitation light by the emission filter in the light-emitting diode (LED) module. The results were displayed on the smartphone screen, as a ratio of a control and test lines on a strip and coordinated with the location on Google Maps, to check for non-/infected areas.

Another group, Wu et al. [77], also have used smartphone assistance for influenza A detection. An automated and portable paper-based microfluidic system was developed. The chip consisted of two modules: the storage module with reagent chambers and the reaction module with the absorbent pad and nitrocellulose membrane functionalized with specific monoclonal antibodies. The smartphone was used for image capturing from the membrane by camera and for processing the image with an algorithm to the application developed with Java. The smartphone was used as a guide to the microcontroller, connected via Bluetooth. It enhanced multiple reaction steps performance, collected the results and sent it to medical agencies if necessary.

Another idea was to use already existing techniques and standardized analyzers. Zhang et al. [78] designed an electrochemical assay which used a glucometer. Glucose-containing substrate (SG1) exposed to influenza virus (or neuraminidase) released glucose which is determined amperometrically. Samples were analyzed directly, without further preparation. The result was the detection of 19 strains of influenza viruses (H1N1 and H3N2) in 1 h. This method offers user-friendly, fast and inexpensive detection.

### 5.3. Electrical-Based Detections

When classifying influenza diagnosis methods by used measurement techniques, dominating are optical [79] and electrical [80]. They are believed to be fast, easy, adequate (providing qualitative and quantitative analysis) and relatively inexpensive.

The influenza virus gold electrode electrochemical sensor was proposed by Horiguchi et al. [81] Immobilization of HA specific receptor (6′-sialyllactose) onto gold allowed label-free H1N1 detection. Quartz crystal microbalance (QCM) technique and electrical detection were developed for biosensor measurements. QCM gave 2^−4^ HAU sensitivity with 10 min detection time and electrical detection gave sensitivity of 2^−6^ HAU and 30 min analysis time. The sensitivity was higher than conventional immunochromatographic technique (ICT).

Different type of bioreceptor, anti-M1 antibodies was developed for influenza A detection. Nidzworski et al. [82] modified boron-doped diamond (BDD) electrodes with 4-aminobenzoic acid and anti-M1 antibodies in self-assembled monolayer (SAM) approach. By electrochemical impedance spectroscopy (EIS) measurements authors achieved the lowest limit of detection (1 fg/mL) compared to previously reported methods. The assay offered easy sample pretreatment, short incubation time (<5 min) and non-interference of bacteria and yeast which might be present in patients swab.

Another antibodies immobilization method was presented by Cheng et al. [83] Simple technique of AC electric field application on electrodes induced positive dielectrophoresis. Subsequently, viral particles were attracted to the immunosensor. Commercially available surface acoustic wave (SAW) electrode chip of 50 mm length size was used. The detection limit was 0.25 pg/mL with very fast response time 30 s. The authors have achieved the sensitivity of 90% and specificity of 70%.

**Figure 3:**
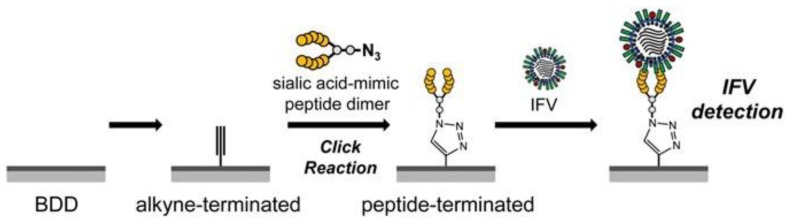
An example of electrode surface modification. Boron-doped diamond electrode modified via click reaction for infectious flacherie virus (IFV) electrochemical detection. Reproduced with permission from Matsubara et al., PNAS; published by PNAS, 2016.

Apart from electrodes mentioned above, there is a wide range of electroactive materials for biomolecule recognition. Available are carbon-based materials like carbon paste, glassy carbon [84,85], graphene-oxide [86], reduced graphene oxide [87], boron-doped diamond (presented in Figure 3) [88].

There are also noble metal-based materials like silver, gold [89,90], platinum, zinc, cadmium, and others [91]. Moreover, every listed material can undergo multiple methods of surface modification, not only with nanoparticles [91,92,93,94,95,96,97], but hybrids and biological materials, like aptamers [98,99,100], sialic acid [88] or its derivatives [101], composites [84,85,102], nanohybrids [103] and dyes [86]. For influenza biosensing the most often used measurement techniques are differential pulse voltammetry [85,91,103], cyclic voltammetry [102], electrochemical impedance spectroscopy [84,104], and amperometry [86,105].

Interesting research of Mubarok et al. [106] showed whole blood analysis for neuraminidase activity label-free detection. The cleavage of glycosidic linkage of self-synthetized *N*-acetyl-2-*O*-(4- aminophenyl)-α-neuraminic acid (AP-Neu5Ac) in the presence of NA released p-aminophenol molecules. As p-aminophenol showed electroactivity, the electrical signal onto a bare glassy carbon (GC) electrode was recorded. LOD of NA activity was 5.6 ng/mL. The sensor was applicable also for urine, saliva and nasal swabs.

Currently, many authors construct glycan-based biosensors [5,19,107] as they are natural viral receptors with selectivity for pathogenic subtypes. They form a compact layer on the measurement surface, so-called glycocalyx, reaching even 100 mM concentration. Simple electrode modification might exclude time-consuming and expensive antibodies assay. For example, LOD for H3N2 was achieved on 13 viral particles per 1 µL level [107]. For glycan-derivatives SAMs the most often used electrochemical techniques are impedance spectroscopy and amperometry.

Except influenza proteins mentioned in ‘2. Influenza pathogenesis’ section, some non-structural proteins PA-X, PB1-F2, NS1, NS2 and others were recently found [108] They were also identified as pathogenic for the host organism [109,110,111]. Electrochemical EIS biosensor for PB1-F2 was developed by Miodek et al. [112] The authors used antibody-antigen approach and modified gold electrode with a specific anti-PB1-F2 antibody with three steps. Firstly, pyrrole and ferrocene derivatives were electrochemically polymerized onto the Au surface. The next step was biotin/streptavidin linkage and the closing step was biotinylated antibodies immobilization. For confirmation of proper sensor modification the surface plasmon resonance (SPR), atomic force microscopy (AFM) and cyclic voltammetry (CV) techniques were used. By differential pulse voltammetry (DPV) two linear ranges were observed, 50–300 nM and 0.5–1.5 mM of PB1-F2 what was probably caused by two specific antibody sites. LOD was on 0.42 nM level.

### 5.4. Optical-Based Detections

From optical/spectral methods the most common are fluorescence, ultraviolet/visible (UV/Vis) spectroscopy, Surface Enhanced Raman Spectroscopy (SERS) [113,114] and others [79].

Recently developed influenza sensor by Liu et al. [115] used fluorescence for neuraminidase detection. The macrocyclic dye substrate (squaraine-derived core blocked on ends with sialic acid) reacted with viral NA releasing the blockages. Subsequently, the free core was encapsulated with macrocyclic tetralactam what caused spectral changes. Red-shift in absorbance and fluorescence emission of squaraine were observed in the NA presence. Results were visible with naked eye. With some optimization, this portable sensor might be used in influenza diagnosis in POC or for effective determination of antiviral inhibitor drugs. The authors claimed possibilities of differentiation for classes of mammalian, bacterial and viral neuraminidase and suitability for quantitative analysis.

Another technique, SERS, was used in magnetic immunosensor for avian influenza detection [116]. SAM approach of 4-mercaptobenzoic acid (4-MBA) molecules chemisorption on the gold nanoparticles (AuNPs) was used for further influenza A IgG antibodies immobilization. The sandwich-type biosensor structure enabled qualitative and quantitative analysis. H3N2 was detected at LOD 10^2^ TCID_50_/mL with linearity range 10^2^–5 × 10^3^ TCID_50_/mL. The assay had a potential for POC use due to time efficiency, sensitivity, and portability.

A similar study by Park et al. [117] has shown SERS-based assay in a lateral flow strip. The principle was exchanging AuNPs from the commercial kit with SERS-active nanotags. This innovation enhanced precision and sensitivity of the Raman signal. The Au-nanotags-influenza-antibodies complex was captured onto an antibodies-modified strip with positive probes giving two red strains (control line and SERS-test tine). Negative probes gave only one response on the control line. LOD was calculated as 1.9 × 10^4^ PFU/mL and this value was one order of magnitude higher than standard colorimetric kits.

A different detection-type, immunochromatographic test (FICT), for avian influenza A was proposed by Yeo et al. [118] The authors have chosen fluorescent Red dye 53 which intensity increased intensity by additional fluorescent phosphor groups linkage. The assay was to europium-based FICT and standard RIDT. Influenza A virus from nasopharyngeal swabs was detected by a portable fluorescent strip reader. Analysis time was only 15 min, the LOD was estimated at 20 HAU/mL, and the linearity range was 20–640 HAU/mL. FICT was 4-times more sensitive than europium-based FICT and 16-times more sensitive than the rapid diagnostic test.

Another use of dye, 3,3′,5,5′-tetramethylbenzidine (TMB), was in colorimetric immunosensor assays. Lin et al. [58] similarly used antibodies in complex with streptavidin/biotin linker, liposome and antigen. Innovative was horseradish peroxidase (HRP) encapsulation in liposome. After the addition of hydrogen peroxide (H_2_O_2_) and TMB to the complex, lysis of liposomes occurred. HRP catalyzed the decomposition of H_2_O_2_ generating ^·^OH radicals which oxidized TMB and gave a color change to the solution. The detection was possible by the naked eye and spectrophotometric technique. LOD of H5N1 was 0.04 ng/mL with linearity from 0.1 to 4.0 ng/mL. Compared to standard ELISA where very small absorbance and visual no color change was observed with the concentration below 4.0 ng/mL, the authors approach showed much more sensitivity.

The modification of TMB-based assay was proposed by Ahmed et al. [119] For spectrophotometric H3N2 virus detection authors have used gold nanoparticles-carbon nanotubes (AuNPs-CNTs) hybrids, which showed the high catalytic activity of TMB oxidation. The complex of AuNPs-CNTs-TMB-H_2_O_2_ in the influenza presence showed a change in color. Blue tone intensity varied depending on the virus concentration. The complex absorbed the light in λ_max_ = 450 nm which was measured using a microplate reader. This method had a limit of detection 385 times lower (3.4 PFU/mL) than conventional ELISA.

Ahmed et al. [120] similarly have used (+)AuNPs in TMB-based method achieving H1N1 virus detection at even lower levels of 10.79 pg/mL and for H3N2 of 11.62 PFU/mL improving sensitivity to 500-times higher than ELISA.

Next optical detection technique Upconversion Luminescence Resonance Energy Transfer (LRET) was used by Ye et al. [121]. The authors detected avian influenza H7 virus subtypes. The biosensor contained donor fluorophores (BaGdF_5_:Yb/Er upconversion nanoparticles, UCNPs) and acceptor fluorophores (AuNPs). LRET measurement was activated by hybridization between complimentary oligonucleotides which were linked to NPs. The target H7 gene was conjugated to acceptor NPs and complimentary genes to donor NPs. Hybridization process decreased the fluorescence of UCNPs and enhanced light absorption of AuNPs. Upconversion spectra were registered after 2 h probes incubation. LOD was 7 pM of the hemagglutinin gene and linear response from 10 pM to 10 nM.

Glycan-based methods mentioned in the electrical assays also have found application in optical biosensors. Zheng et al. [122] used glycan-functionalized gold nanoparticles (gGNPs) that bounded and aggregated on the viral surface. The authors have differentiated fourteen influenza strains and distinguished them from a human respiratory syncytial virus. The principle was different HA-binding preferences depending on the configuration of C-C bond in sialic acid receptors (described in Section 2. ‘Influenza pathogenesis’). The one-step procedure, mixing virus with gGNPs and 90 min incubation resulted in a color change from red to purple and was measured spectrophotometrically.

Except for natural glycans, He et al. [123] have synthesized influenza virus NA resistant sialosides, with C-, S- and triazole linkage ends and printed onto a glass surface. These molecules could capture eight virus strains at very low concentration. Caught viruses gave fluorescence intensity rise. LOD was on 35 CEID_50_ (Chicken Embryo Infectious Dose).

Adegoke et al. [124] as first evaluated the synthesis of CdZnSeTeS Quantum Dots (QD) with the one-pot hot-injection method. They were used for near-infrared-emitting viral RNA detection. The fluorescence signal was measured after 3 min incubation of QD and H1N1 probe. Low LOD level (1 copy/mL) confirmed much better sensitivity than RIDTs.

### 5.5. Modifications of Standard Methods

The standard influenza virus detection method (RT-PCR) was modified by Hmila et al. group [125]. The novel assay development was motivated by new mutations in HA and NA of Tunisian poultry. The risk of economic losses and human infections significantly increased. The authors have used an aptamer-real time-PCR. Advanced was using one step SELEX procedure for H9N2 specific ssDNA aptamers selection and later use as ligands for virus capture. The assay was adequate for direct swabs with no need for sample pretreatment and showed rapid, label-free results and high sensitivity. Conventional ELISA showed LOD on 1.00 × 10^5^ TCID_50_/mL and this assay 1.00 × 10^2^ TCID_50_/mL.

Another group [126] has performed a new platform in China where standard PCR was conjugated with mass spectrometry-electrospray ionization (ESI-MS) for identification of respiratory viruses. The authors have used nasopharyngeal aspirates for the analysis and have compared their method with DFA and later confirmed by RT-PCR plus sequencing. PCR-ESI-MS showed higher sensitivities because detected more viruses in patients’ specimens and was adequate for co-infections determination. The method could detect many virus families like coronaviruses, adenoviruses, alphaviruses and others. PCR-ESI-MS was believed as faster and more automatic than conventional FDA but required expensive equipment and had no practical usage in POC devices.

For rapid and accurate influenza detection with high sensitivity and short time, Eboigbodin et al. [127] have proposed the assay combining two techniques. Reverse transcription (RT) of cDNA to RNA in single-step reaction and strand invasion-based amplification (SIBA^®^) in isothermal conditions allowed to detect both influenza A and B in only 15 min where traditional RT-PCR required more than 50 min. Beneficial was RNA usage for detection instead of DNA as it is major influenza genetic material. 100 copies of H1N1 RNA were detected with 100-times higher sensitivity than RT-PCR.

Modification of LAMP method was proposed by Ge et al. [128] LAMP is believed to be the most willingly used nucleic acid-based isothermal amplification assay. However, differentiation of products containing multiple targets is still challenging as currently existing methods (e.g., electrophoresis) require specialized equipment. Simple colorimetric method with invasive gold nanoparticles reaction was used. NPs aggregation and dispersion resulted in solution color change. The sensor could subtype H1 an H3 with LOD of 10 RNA copies and influenza B with LOD 100 RNA copies.

Coordination of two trending viral detection methods, electrical and optical was proposed by Sepunaru et al. [129] Virus tracking was achieved by UV-Vis spectroscopy. Influenza particles were able to absorb silver NPs and showed maximum absorbance at 401 nm. Moreover, the tagged virus could be absorbed on the GC electrode and gave an electrical response. Chronoamperometric signal of silver NPs oxidation process was proportional to virus quantity. This assay enabled viral and bacterial infection distinction.

Sakurai et al. [130] have improved the common antigen-detection rapid influenza test known as immunochromatography (IC). IC could be accomplished in less than 20 min, however, had low sensitivity (around 60%) and LOD on 10³ PFU which is lower than PCR-based methods. The improvement was based on antibodies conjugation with fluorescent beads what enhanced the sensitivity 100-times. Moreover, the assay was more accurate for early infections detection than IC.

### 5.6. Other Novel Ideas

**Figure 4:**
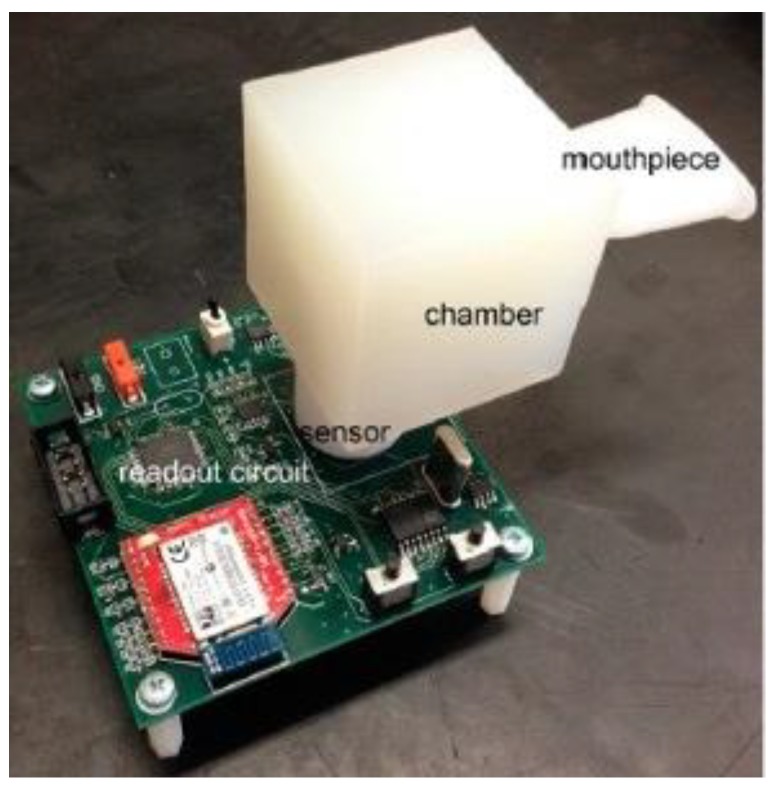
Novel Isoprene Sensor readout with Bluetooth mode. Reproduced with permission from Gouma et al., Sensors; published by MPDI, 2017.

Gouma et al. [131] have recently invented the Novel Isoprene Sensor for an influenza virus (Figure 4). The authors have claimed that infected patients generate more volatile products compared to healthy ones. Volatile products come from the alveolar and airway epithelium as well as leukocytes infiltrating the lungs, like volatile organic compounds (VOCs) and nitric oxide (NO). They were used as biomarkers to detect the disease. The constructed device was a portable 3-sensor array microsystem offering rapid non-invasive screening. The measurement needed to be conducted as fast as the disease was (potentially) present to observe biomarkers changes in time. The sensor was believed to give satisfying specificity and sensitivity. More precisely, the sensor could detect three gases: isoprene, ammonia, NO in the temperature control conditions. It measured resistance changes of h-WO_3_ material with exposure to NO, NO_2_, methanol, and isoprene at 350 °C.

Jiang et al. [132] have invented an influenza sensor using a SAW platform. The piezoelectric LiNbO_3_ wafers were coated with SiO_2_. In this technique, the propagation of acoustic waves was changing at the measured material, depending on analytes located on the surface. Love waves detected antigens through specific antigen-antibody interaction. The authors have used two reagents that were effective for immobilizing HA antibodies on the measurement surface. They were triethoxysilybutyladehyde (ALTES) and triethoxysilylundecanal ethylene glycol acetal (ACTES), which were end-functionalized with carboxylic acid and aldehyde. LOD for H1N1 HA antigen was 1 ng/mL.

Oyama et al. [133] have developed their point-of-care testing chip. It was based on antigen-antibody interactions with fluorescence detection. The authors applied absorbing polymer providing continuous sample flow and separation of bounded and free antibody residues. A glass fiber sheet was chosen as a flow medium. Antibodies were fluorescently labeled by Dylight488 and Dylight650. The achieved sensitivity was more than thousand times better compared to immunochromatographic commercial assays (LOD above 104 ng/mL).

Krishna et al. [134] have developed giant magnetoresistance (GMR) sensor, which adapted monoclonal antibodies to influenza H3H2v nucleoprotein with magnetic nanoparticles (MNPs). In the virus presence, MNPs were bonded to the sensor, and the resistance change was measured. LOD was 1.5 × 10^2^ TCID_50_/mL. This assay was applicable for nasal swabs.

Except for the methods mentioned above, many authors have proposed novel ideas. For example, Shelby et al. [18] invented the magnetic relaxation nanosensor (MRnS) which was selectively binding to hemagglutinins. The influenza virus variants detection was at 1.0 nM concentration level. Kirkegaard et al. [135] invented screen-printed aptasensor (PEDOT) for impedance-based influenza A detection. Ozcelik et al. [136] evaluated the optofluidic wavelength division multiplexing method for single-virus detection. The authors could distinguish three subtypes of influenza A using two approaches. First was virus labeling with different color labels, and second was the combination of two different colors for every viral strain. Lee et al. [137] presented quantitative H7N9 virus screening without DNA amplification, basing on single-particle dual-mode total internal reflection scattering (SD-TIRS) with transmission grating (TG). Chan et al. [138] claimed that the flow graphene transistor-based DNA sensors have not been explored yet, so they proposed microfluidic integrated reduced graphene oxide (rGO) transistor for the H5N1 influenza virus gene detection. Zhang et al. [139] elaborated a label-free optical sensor for influenza serotyping. The authors have used the pattern recognition method based on Arrayed Imaging Reflectometry (AIR) platform. When antireflective chip condition was perturbed due to binding to, e.g., an antibody, the reflected light changes were quantitatively detected. Katayama et al. [140] created an influenza virus biosensor based on two methods: electrochemiluminescence (ECL) of modified Au electrode combined with immunoliposome encapsulating Ru (II) complex. The authors have achieved sensitivity higher than ELISA; the detection range was from 2.7 × 10^2^ to 2.7 × 10^3^ PFU/mL. Li et al. [141] combined complement fixation and luminol chemiluminescence for rH7N9 detection, which is a recombinant avian influenza virus protein. This assay could be completed in 2.5 h with a linear detection from 0.25 fg/mL to 25 ng/mL. Tran et al. [142] detected influenza A viruses using carbon nanotubes field effect transistor (CNTFET) DNA sensor. The response time was less than 1 min, and the detection range was linear from 1 pM (LOD) to 10 nM. The sensor signal recovery was 97% after 7-month storage in pH-controlled conditions. The most interesting biosensors were compared in Table 2.

## 6. Conclusions

The scope of this article was presenting progress in the influenza sensing area, discussing the chosen materials and recognition elements, measurement techniques, sensitivities, specificities and further applications in POC devices.

From all the presented influenza virus diagnosis methods, the most used in laboratories are ‘gold standard’ conventional methods. However, the majority of these require long time analysis (6–24 h for RT-PCR) [40] but compensating it with high sensitivities and the most reliable results. That is why the need for improvement for ‘gold standards’ increase significantly.

Despite the fact of low and variable sensitivity, RIDTs are the main improvement in influenza diagnostics, especially in pandemic seasons. They are pivotal in the Emergency Rooms by decreasing numbers of unnecessary antibiotics dosages. Performing RIDTs at physicians’ offices relieves laboratories work and enables the focus on specimens designed for culture or other time-consuming methods. Moreover, the increased interest in specific laboratory diagnosis is due to minimizing the antibiotics resistance of patients and improves the influenza recognition system.

As influenza is a public-health threat and influenza A is pandemic, portable, fast and accurate tools are in high demand in the medical industry to control virus outbreaks and spreads. Also, this kind of devices needs constant updates due to genetic assortment of human, swine and avian influenza. New H/N genes are produced, against which human population lacks immunity.

Generally understood the ‘biosensors’ field has attracted many scientists. From a wide range of proposed assays, it is possible to choose these with reasonable cost, good selectivity and sensitivity, and practical application in POC. For authors of this article, the electrochemical sensors win the competition. It seems that they meet the need for rapid and accurate influenza diagnosis. They offer a vast number of electrode materials and target detection molecules with practically endless modification methods. Furthermore, these sensors successfully can be expanded for other pathogen detection by changing the kind of probe immobilized on the electrode surface.

## Figures and Tables

**Table 1:** Conventional methods of influenza virus detection.

Rapid Diagnostic Tests	Immunofluores-Cence Assays	Serological Assays	Cell Culture Based Detection	Nucleic Acid-Based Tests	Next-Generation Sequencing Based Methods
Alere ^TM^ and Influenza A&B	direct fluorescent antibody staining	hemagglutina-tion inhibition	Immunofluores-cence microscopy	reverse transcriptase-PCR	Sanger method
BD Veritor ^TM^	indirect fluorescent antibody staining	virus neutralization	antibody staining	sequencing-based tests like NGS	Illumina Platform
Sofia^®^ Influenza A + B		single radial hemolysis	erythrocytes hemadsorption	ligase chain reaction	Roche 454 Life Sciences
the cobas^®^ Liat^®^ Influenza A/B		complement fixation		DNA microarray tests	Pacific Bioscience
BUDDI^TM^ Influenza		ELISA		SAMBA	Ion Proton
Quick Navi-Flu^TM^		Western blotting		NASBA	Complete Genomics
				LAMP	Luminex

**Table 2:** Comparison of novel detection methods of influenza virus.

Detection Method	Target Molecule	LOD	Linear Range	Detection Time	Reference
**Microchip approaches**					
microfluidic RT-PCR + DEP chip	DNA	5.36 × 10^3^ copies/mL	5.36 × 10^3^–5.36 × 10^5^ copies/mL	15 min	[73]
**Reuse of known devices**					
smartphone-based fluorescence	VP	7.5 PFU/mL	0.94 × 10^0^–4.8 × 10^2^ PFU/mL	15 min	[76]
**Electrical-based detections**					
EIS	M1	1 fg/mL	1–100 fg/mL	5 min	[82]
dielectrophoresis	VP	0.25 pg/mL	1:100 000 dilution factor	30 s	[83]
LSV	NA	5.6 ng/mL	0–900 ng/mL	30 min	[106]
DPV	PB1-F2	0.42 nM	50–300 nM; 0.5–1.5 mM	-	[112]
**Optical-based detections**					
spectrophotometry	VP	0.04 ng/mL	0.1–4.0 ng/mL	30 min	[58]
SERS + LFA	VP	1.9 × 10^4^ PFU/mL	0–1.0 × 10^6^ PFU/mL	-	[117]
LRET	HA	7 pM	10 pM to 10 nM	2 h	[121]
NIR	RNA	1 copy/mL	0–14 copies/mL	3 min	[124]
**Modification of standard methods**					
RT-SIBA	RNA	100 copies	-	<30 min	[127]
mRT-LAMP-CIRN	RNA	10^1^/10^2^ copies	10^4^–10^0^ RNA copies/μL	~30 min	[128]
**Other novel ideas**					
SAW	HA	1 ng/mL	1–100 ng/mL	10 min	[132]
GMR	NP	1.5 × 10^2^ TCID_50_/mL	1.5 × 10^2^–1.0 × 10^5^ TCID_50_/mL	60 min	[134]
SD-TIRS + TG	DNA	74 zM	74 zM–7.4 fM	50 ms	[137]
conductance	DNA	5 pM	10 pM–100 nM	1 h	[138]
ECL	HA-P	2.7 × 10^2^ PFU/mL	2.7 × 10^2^–2.7 × 10^3^ PFU/mL	1 h	[140]
CFT + CL	RP	0.14 fg/mL	0.25 fg/mL–25 ng/Ml	2.5 h	[141]
CNTFET	DNA	1 pM	1 pM–10 nM	2 h	[142]

VP—viral particles, NIR—near-infrared fluorescence, HA-P—hemagglutinin peptide, RP—recombinant protein, LSV linear sweep voltammetry, SERS + LFA-surface-enhanced Raman scattering—based lateral flow assay, RT-SIBA—reverse transcription strand invasion-based amplification, mRT-LAMP-CIRN—reverse transcription loop-mediated isothermal amplification-based assay coupled with cascade invasive reaction, CFT + CL—complement fixation test+ luminol chemiluminescence.
